# A Self-Setting Hydrogel of Silylated Chitosan and Cellulose for the Repair of Osteochondral Defects: From *in vitro* Characterization to Preclinical Evaluation in Dogs

**DOI:** 10.3389/fbioe.2020.00023

**Published:** 2020-01-29

**Authors:** Cécile Boyer, Gildas Réthoré, Pierre Weiss, Cyril d’Arros, Julie Lesoeur, Claire Vinatier, Boris Halgand, Olivier Geffroy, Marion Fusellier, Gildas Vaillant, Patrice Roy, Olivier Gauthier, Jérôme Guicheux

**Affiliations:** ^1^Inserm, UMR 1229, RMeS, Regenerative Medicine and Skeleton, Université de Nantes, ONIRIS, Nantes, France; ^2^Université de Nantes, UFR Odontologie, Nantes, France; ^3^CHU Nantes, Service d’Odontologie Restauratrice et Chirurgicale, PHU4 OTONN, Nantes, France; ^4^SC3M – “Electron Microscopy, Microcharacterization and Functional Morphohistology Imaging” Core Facility, Structure Fédérative de Recherche Franc̨ois Bonamy, INSERM – UMS016, CNRS 3556, CHU Nantes, Université de Nantes, Nantes, France; ^5^CHU Nantes, PHU4 OTONN, Nantes, France; ^6^Centre of Research and Preclinical Investigation (C.R.I.P.), ONIRIS, Nantes, France

**Keywords:** hydrogel, cell therapy, cartilage, regenerative medicine, osteoarthritis

## Abstract

Articular cartilage (AC) may be affected by many injuries including traumatic lesions that predispose to osteoarthritis. Currently there is no efficient cure for cartilage lesions. In that respect, new strategies for regenerating AC are contemplated with interest. In this context, we aim to develop and characterize an injectable, self-hardening, mechanically reinforced hydrogel (Si-HPCH) composed of silanised hydroxypropymethyl cellulose (Si-HPMC) mixed with silanised chitosan. The *in vitro* cytocompatibility of Si-HPCH was tested using human adipose stromal cells (hASC). *In vivo*, we first mixed Si-HPCH with hASC to observe cell viability after implantation in nude mice subcutis. Si-HPCH associated or not with canine ASC (cASC), was then tested for the repair of osteochondral defects in canine femoral condyles. Our data demonstrated that Si-HPCH supports hASC viability in culture. Moreover, Si-HPCH allows the transplantation of hASC in the subcutis of nude mice while maintaining their viability and secretory activity. In the canine osteochondral defect model, while the empty defects were only partially filled with a fibrous tissue, defects filled with Si-HPCH with or without cASC, revealed a significant osteochondral regeneration. To conclude, Si-HPCH is an injectable, self-setting and cytocompatible hydrogel able to support the *in vitro* and *in vivo* viability and activity of hASC as well as the regeneration of osteochondral defects in dogs when implanted alone or with ASC.

## Introduction

Articular cartilage (AC) is a complex porous permeable extracellular matrix that surrounds specific cell type called chondrocytes. Due primarily to its avascular and aneural structural organization (Clouet et al., 2009), AC does not heal spontaneously when injured. AC defects affect approximately two million patients every year in Europe and in the United States (Mumme et al., 2016), and they remain a clinical challenge due to the paucity of routinely translatable therapeutics to treat this condition. Degenerative changes affected the cartilage and the bone surrounding the lesion (Schinhan et al., 2012). Therapeutic options to repair AC defects include microfracture, autologous osteochondral grafting (Brittberg et al., 2016), and autologous chondrocyte implantation. These therapeutic options are, however, subject to major logistical and clinical issues, such as their short-lived clinical benefits in particular (Vinatier and Guicheux, 2016).

Articular cartilage defects, when left untreated or partially healed, dramatically increase the predisposition to osteoarthritis (OA). OA is a painful, debilitating, and slowly progressing degenerative and inflammatory joint disease that is initiated by cartilage damage prior to its culmination in alteration of all of the joint tissues (i.e., synovium, bone, muscle, tendon, and ligament) (Hunter and Bierma-Zeinstra, 2019). As there is no curative treatment, prosthetic joint replacement remains the last-recourse for alleviation of OA-associated pain symptoms (Vinatier et al., 2016). In light of this, a number of innovative strategies to repair or regenerate cartilage and subchondral bone very early in the degenerative joint cascade have been proposed. Among these strategies, tissue engineering (TE) is thought to be one of the most promising approaches (Makris et al., 2015). It consists primarily of combining reparative cells with a biomaterial capable of supporting cell transplantation as well as their engraftment, viability, growth, differentiation, and secretory activity.

Cartilage tissue engineering was initially focused on the use of chondrocytes of articular origin (Brittberg et al., 1994). However, the difficulties encountered, particularly with loss of the chondrocyte phenotype during their *in vitro* expansion phase and the morbidity of the donor site (Benya and Shaffer, 1982; Vinatier et al., 2009a), has led researchers to explore new cell sources. Nasal chondrocytes have been proposed as an alternative to articular chondrocytes (Vinatier et al., 2007), and their successful clinical use in association with a collagen membrane was recently reported (Mumme et al., 2016). Another alternative to articular chondrocytes that has been widely investigated is adult mesenchymal stromal cells (MSC). MSC can readily be isolated from various tissues such as bone marrow (BMMSC) (Friedenstein et al., 1968), adipose tissue (ASC) (Fraser et al., 2006), and synovial fluid (SFAC) (Jones et al., 2004). They have the capacity for self-renewal and differentiation into a range of cell types encompassing chondrocytes in particular, as well as other cell types of the musculoskeletal system (Pittenger, 1999). In addition, recent studies have shown that MSC are able to secrete a myriad of biological factors, either directly or through the release of extracellular vesicles (Heldring et al., 2015). These cytokines, chemokines, and growth factors can exert immunomodulatory effects (Le Blanc and Davies, 2015), reduce tissue damage, and promote repair or healing processes. Finally, as MSC generally fail to express the class two major histocompatibility complex molecules, they may be candidates for allogeneic transplantation without host alloreactivity (Ankrum et al., 2014) and hence of particular clinical interest for cartilage TE. This immune privilege of MSC, although it is still a matter of considerable debate (Ankrum et al., 2014), paves the way of the development of “off-the-shelf” MSC therapies.

A number of biomaterials have been proposed in recent years as cell carriers for cartilage and subchondral bone TE (Morris et al., 2010). Among these scaffolding biomaterials, hydrogels have been widely investigated in light of their favorable physicochemical and biological properties (e.g., high water content, biocompatibility, tunable mechanical properties, and permeability) (Hoffman, 2001; Guan et al., 2017). With the aim of developing a self-crosslinking hydrogel, we have devised and patented injectable silanized hydroxypropylmethyl cellulose (Si-HPMC). Si-HPMC was initially used for the 3D culture of MSC and chondrocytes (Vinatier et al., 2005, 2009b; Merceron et al., 2011). More recently, Si-HPMC has also been successfully used for MSC based regeneration of soft tissues such as myocardium (Mathieu et al., 2012) and colon (Moussa et al., 2017). It has also been preclinically tested, albeit with less encouraging results, for the repair of stiffer tissues such as cartilage (Vinatier et al., 2007; Portron et al., 2013). Interestingly, a converging body of proof has recently indicated that MSC are “touchy-feely” cells that are particularly able to sense the biomechanical properties (e.g., stiffness, elasticity, and relaxability) of their micro-environment (Discher et al., 2009). When cultured on stiff materials, they preferentially engage in osteochondral differentiation, while on soft materials they commit to brain or cardiac differentiation pathways (Engler et al., 2006; Murphy et al., 2014). In light of these data, there appears to be ample merit in developing hydrogels for cartilage TE that have mechanical properties that mimic those of the target tissue. To address this issue, we sought to determine whether adding mechanical reinforcement to Si-HPMC may be a viable strategy to develop materials that not only have improved mechanical properties but also increased cartilage regenerative capacity. In light of the silanol-dependent reticulation process of Si-HPMC and our ability to silanize a large panel of biomolecules including polysaccharides (patent WO2011089267), we have synthesized a hybrid hydrogel of Si-HPMC and Si-chitosan. Chitosan is a natural chitin-derived polymer extracted from the exoskeleton of crustaceans. It is composed of D-glucosamine and N-acetyl-D-glucosamine (Oprenyeszk et al., 2015), which can mimic the glycosaminoglycan content of cartilaginous extracellular matrix (Lahiji et al., 2000; Hoemann et al., 2007). Several studies have also shown that chitosan enhances the viability of bovine chondrocytes (Sechriest et al., 2000) and the chondrogenic differentiation of MSC (Cho et al., 2004; Dang et al., 2006) in culture. In addition, chitosan has mucoadhesive properties that are useful for scaffold anchorage to native cartilage (de Campos et al., 2005). Chitosan is also positively charged (Morris et al., 2010), sterilizable, biocompatible (Debnath et al., 2015), and biodegradable (Lee and Mooney, 2001). All of these characteristics make chitosan an appropriate candidate for silanization and further combination with Si-HPMC for use in cartilage TE.

The aim of this study was to develop and characterize a new mechanically reinforced hybrid hydrogel made of Si-HPMC and Si-chitosan (referred to here as Si-HPCH) and to evaluate its ability to repair osteochondral defect in a dog model. We first characterized the mechanical and rheological properties of Si-HPCH. The viability of human MSC was then assayed after *in vitro* culture either in contact with or embedded in Si-HPCH. The viability of human MSC was also investigated after transplantation with Si-HPCH in the subcutis of nude mice. Lastly, we assessed the preclinical potential of Si-HPCH in cartilage repair using a canine model of osteochondral knee defects.

## Materials and Methods

### Si-HPMC and Si-Chitosan Synthesis and Hydrogel Preparation

Silanized hydroxypropyl methylcellulose and an acidic buffer solution (ABS, pH 3.6) were prepared as previously described (Bourges et al., 2002a, b). Silanized chitosan (Si-chitosan) was synthesized as follows. One hundred milliliters of chitosan solution (1% wt/v, HCl, pH 3.0) was poured into a round bottom flask and 1 eq (1.55 mL) of (3-Isocyanatopropyl)triethoxysilane was added. The mixture was then stirred for 3 h at room temperature and then initially dialyzed (molecular weight cut-off of 6–8 kDa) for 2 days against NaOH (0.1 M) with frequent replacement of the dialysis solution. The sample was then dialyzed against water with frequent replacement of the dialysis solution until the conductimetry monitoring indicated no further release. The dialyzes eliminated the non-grafted silane derivatives used for siloxane grafting onto the HPMC and chitosan. As a final step, the purified gels were lyophilized.

Two types of hydrogel were prepared. The first one was made of Si-HPMC alone and prepared by dissolving Si-HPMC polymer (3 wt%) in 0.1 M NaOH aqueous solution. The second one, referred to as Si-HPCH, was composed of Si-HMPC and Si-chitosan. It was prepared as follows: Si-HPMC polymer (3 wt%) was dissolved in 0.1 M NaOH aqueous solution; Si-chitosan (3 wt%) was then dissolved in the 3 wt% Si-HPMC aqueous solution (0.1 M NaOH).

The hydrogel precursor solution was then obtained by mixing 1 volume of the above Si-HPMC or Si-HPCH basic solution contained in a Luer lock syringe, with a 0.5 volume of an ABS prepared in our laboratory (Bourges et al., 2002a, b) at pH 3.6 in another Luer lock syringe, by interconnection of both syringes; the final pH of the mixture was 7.4. This precursor mixture was injectable for 30–40 min, at which time the gel point was reached.

### Mechanical and Rheological Characterization

The viscoelastic modulus (storage modulus G′) and the breaking strength (σ) were obtained using a HAAKE MARS rheometer (Thermo Fisher Scientific, United States) using a plate geometry (PP20Ti, titanium plateau with a 20 mm diameter). Immediately after mixing, the liquid hydrogel precursor solutions were injected into a mold (20 mm in diameter and 5 mm in height) and the measurements started 5 days later (full cross-linking of the network). The storage modulus (G′) and the breaking strength (σ) were monitored as a function of strain (from 0.1 to 3,000 Pa) at a constant frequency and temperature (1 Hz and 23°C). Each condition was measured in quintuplicate from 2 different batches of both hydrogels.

Rheological measurements were performed using a Haake MARS rheometer (ThermoHaake^®^, Germany) with a titanium cone-plate geometry (60 mm in diameter, 1°cone angle, 52 μm gap). Steady shear tests were carried out at 23°C on Si-HPMC (2 wt%) and Si-HPCH (4 wt%) solutions. The operating shear rate ranged from 0.1 to 100 s^–1^. Different flow curves were fitted and extrapolated to lower shear rates by the Cross equation (Cross, 1969). The injectability properties were investigated using a compression testing device (TAHDplus) with a 5 kg load cell for the measurements at an injectability rate of 2 mm per second through a 3 mL syringe equipped with an 18G needle. A syringe containing 1 mL Si-HPMC and Si-HPCH was set and the injection force was measured.

### Cell Viability

#### Adipose Stromal Cell (ASC) Culture

Adipose Stromal Cell were obtained from human patients (hASC) undergoing liposuction and who had provided written consent (ethics committees: Agence de BioMedecine no PFS08-018, the legislation code L.1211-3 to L.1211-9: residues obtained during a surgical procedure, performed in the interest of the person operated, can be used for scientific research), or from autologous canine adipose tissue (cASC) harvested from the gluteal area (APAFIS #4446). Briefly, and as previously described (Merceron et al., 2009), human lipoaspirate and canine adipose tissue were shredded into small pieces and washed extensively with Hanks’ balanced salt solution (HBSS) to remove debris. The washed adipose tissue was treated with collagenase (0.025%) in HBSS for 1 h at 37°C under gentle agitation. The collagenase (Sigma-Aldrich C2674) was inactivated by the addition of an equal volume of Dulbecco’s modified Eagle’s medium (DMEM-Glutamax; Gibco) supplemented with 1% penicillin/streptomycin (P/S) containing 10% fetal calf serum (FCS; control medium). The digested product was then centrifuged at 250 × g for 5 min to separate the adipose fraction from the stromal fraction. The supernatant was removed, and the stromal cells were resuspended in the control medium and filtered through a 70 μm nylon mesh filter. The filtrate was centrifuged and the cells were resuspended in red blood cell lysis buffer. The lysis reaction was stopped by the addition of control medium. The suspension was centrifuged, and the cells were finally resuspended in control medium and plated at a density of 5 × 10^4^ cells/cm^2^.

Adipose Stromal Cell isolated using the protocol described above have been extensively characterized in our laboratory (for details see Merceron et al., 2009, 2011). The medium was replaced 24 h after seeding to remove non-adherent cells. To prevent spontaneous differentiation, primary cultures (P0) of ASC were grown to approximately 80% confluency and then detached from the cell culture flask using trypsin-EDTA. All of the culture incubations were performed at 37°C in a humidified atmosphere containing 5% CO_2_, and the medium was replaced every 2 days. The different experiments with hASC and cASC were carried out with cells at passage 2.

#### Cell Viability in 2D

Human adipose stromal cells (*n* = 3) and cASC (*n* = 1) viability was evaluated by the methyl tetrazolium salt (MTS) assay (Promega, United States). The cells were cultured in physical contact with Si-HPMC or Si-HPCH. hASC and cASC were seeded onto culture plates and were allowed to attach to 48-well plates at a final density of 10,000 cells per cm^2^. After 24 h, the culture medium was removed and either Si-HPMC or Si-HPCH hydrogel (200 μL/well) was added on top of the cell layer. After 1 h of gelation at 37°C, 200 μL of culture medium was added to each well and replaced every 2 days. As a negative control, cells were cultured in the presence of actinomycin-D (5 μg/mL), a well-known inducer of cell death. Finally, the MTS assay was performed on days 0, 1, and 7 for the hASC and on days 0 and 7 for the cASC. The MTS assay is based on the reduction of MTS tetrazolium compound by viable cells, thereby generating a colored and soluble formazan product in the culture medium. The amount of the colored product was determined by the optical density reading at 490 nm (Victor3V 1420 Multilabel Counter). Each condition was tested in triplicate from three different donors of hASC and one donor of cASC.

#### Cell Viability in 3D

3D cell viability was evaluated by the Live/Dead Cell Viability assay (Thermo Fisher Scientific, MA, United States) using hASC (*n* = 3) or cASC (*n* = 1) cultured three-dimensionally in hydrogels at a final density of 1 × 10^6^ cells per mL of hydrogel. The ASC were collected and gently mixed with Si-HPMC or Si-HPCH. Cellularized hydrogels were molded and allowed to gelate in the wells of a 48-well plate at 37°C for 1 h. After gelation, 200 μL of culture medium were added to each well and replaced every 2 days (Vinatier et al., 2007). As a negative control, cells were cultured in 3D in Si-HPMC hydrogel in the presence of actinomycin-D (5 μg/mL). A Live/Dead Cell Viability assay was performed according to the manufacturer’s instructions on days 0, 1, and 7 for hASC and on days 0 and 7 for cASC. Green fluorescence can be observed in the living cells due to the conversion of non-fluorescent calcein AM into green-fluorescent calcein by the intracellular esterase activity in live cells. Dead cells become red-fluorescent due to the uptake of ethidium homodimer-1 as a result of the loss of plasma membrane integrity. The red and green fluorescence were observed with a confocal microscope [Nikon D-eclipse C1 (Ar/Kr)]. The numbers of green and red cells were quantified with Volocity software and the results shown as the percentage of living cells. Each condition was tested in triplicate from three different donors of hASC and one donor of cASC.

### *In vivo* Experiments

#### Nude Mice Subcutis

To investigate whether our biomaterial was able to support the viability of cells *in vivo*, a single-cell suspension of 1 × 10^6^ hASC was gently mixed with Si-HPMC or Si-HPCH hydrogel prior to subcutaneous implantation in 7-week-old female Swiss nude mice (Charles River, L’Arbresle, France), as described previously (Vinatier et al., 2009b; Merceron et al., 2011). All of the animals were treated in accordance with the Medical Animal Care Guidelines of Nantes University (APAFIS#4213). General anesthesia was achieved in an induction chamber with isoflurane (2%) delivered in O_2_ and maintained through an individual mask. The Si-HPMC and Si-HPCH cellularized hydrogels were injected subcutaneously in the back along each side of the dorsum of nude mice. The implantations were performed under aseptic conditions. The two different conditions (“Si-HPMC” and “Si-HPCH”) were tested (*n* = 4), four animals received implants (two implants per animal). The animals were sacrificed 6 weeks after implantation. The mice were euthanized by an overdose of isoflurane in an induction chamber. The hydrogels were individually explanted and the samples were processed histologically as described below.

#### Canine Osteochondral Defects

To investigate whether or not Si-HPCH biomaterial associated with autologous ASC was able to support the repair of osteochondral defects, we undertook a preclinical experiment in dogs. All animal handling and surgical procedures were conducted according to European Community guidelines for the care and use of laboratory animals (EU Directive 2010/63), after approval by the Pays de la Loire Animal Ethical Committee (APAFIS #4446) and the Oniris College of Veterinary Medicine Animal Welfare Committee. Surgical procedures were performed with the animals under general anesthesia and under surgical aseptic conditions.

Self-hardening, mechanically reinforced hydrogel was implanted into calibrated 6 mm-diameter osteochondral defects. Twelve clinically healthy adult Beagle female dogs with body weights ranging from 10 to 15 kg, split into two groups, were used in this study. Osteochondral defects were generated using a 6-mm in diameter orthopedic bur to create a calibrated 6 mm × 5 mm deep osteochondral defect.

A total of 14 defects were created on the medial femoral condyles, one defect per dog (except for two dogs that had two defects, one on each femur). The defects of six dogs were filled with Si-HPCH alone (the Si-HPCH group), while the defects of the six other dogs were filled with Si-HPCH associated with autologous ASC (2 × 10^6^ cells/mL; the Si-HPCH/cASC group). In an attempt to reduce the number of animal lives, a second defect was generated on the contralateral limb in two dogs of the Si-HPCH group and left empty (the Empty Defect group). These two empty defects were used as critical-sized controls. The dogs were sacrificed 4 months after the implantation using intravenous overdose of pentobarbital. Femoral extremities were immediately dissected and prepared for further histological analysis.

### Histological Analyses

Subcutaneous samples were fixed in 10% formalin and embedded in paraffin. Embedded samples were stained as described previously (Vinatier et al., 2009b; Merceron et al., 2011). Briefly, the paraffin-embedded explants were cut into 5 μm-thick sections passing through the middle of the sample. The sections were deparaffinized by immersion in methylcyclohexane, rehydrated by a graded series of ethanol, and then rinsed in distilled water. The tissue sections were stained with Alcian blue (AB). The sections were then visualized and scanned using a NanoZoomer device (Hamamatsu Photonics, Japan).

Canine femoral extremities were fixed in 10% formalin and embedded in resin (Tecknovit^®^ 9100, Heraeus Kulzer, Japan). The embedded samples provided 7 μm thick sections according to the osteochondral defect axis. The resin sections were then deplastified using acetone, rehydrated by a graded series of ethanol, and rinsed in distilled water. The tissue sections were stained with hematoxylin-eosin (HE), Safranin-O (SO), or Movat pentachrome (Movat). For IHC, the sections were incubated with primary antibodies against type II (MP Biomedicals 08631711, United States) or type I (Abcam ab6308, United Kingdom) collagen. The primary antibodies were detected using a kit (DAKO Agilent, Agilent Technologies, United States) with DAB substrate as per the manufacturer’s instructions. The sections were then visualized and scanned using a NanoZoomer device (Hamamatsu Photonics, Japan).

To quantify the histological repair of osteochondral defects, we used a modified version of the grading scale described by O’Driscoll et al. (1986). This histological scoring was based on 10 histological parameters (Table 1). We assigned a score ranging from 0 to 4 points to each criterion for a total of 40 points. Three investigators with extensive experience in cartilage histological analyses performed a blind rating of the stained sections.

**TABLE 1:** Histological parameters used for O’Driscoll-based quantitative scoring of cartilage repair.

Histological parameters	
**Degree of filling**	
– 80 to 100%	4
– 60 to 80%	3
– 40 to 60%	2
– 20 to 40%	1
– 0 to 20%	0
**Surface regularity**	
– Flush	4
– Rough	3
– Slight depressed	2
– Depressed	1
– Overgrown	0
**Glycosaminoglycan content**	
– Normal	4
– Nearly normal	3
– Moderate	2
– Weak	1
– None	0
**Cartilage thickness**	
– Similar to the surrounding cartilage	4
– Greater than the surrounding cartilage	3
– Less than the surrounding cartilage	2
– Very thin layer of cartilage	1
– No cartilage	0
**Cellular morphology**	
– Hyaline cartilage	4
– Mostly hyaline cartilage	3
– Mostly fibrocartilage	2
– Fibrocartilage	1
– No cartilage	0
**Type 2 collagen staining in the defect**	
– Normal	4
– Nearly normal	3
– Moderate	2
– Slight	1
– None	0
**Type 1 collagen staining in the defect**	
– None	4
– Slight	3
– Moderate	2
– Important	1
– Complete	0
**Adjacent cartilage quality**	
– Normal	4
– Nearly normal	3
– Moderate	2
– Poor	1
– Degraded	0
**Subchondral bone integrity**	
– Normal	4
– Nearly normal	3
– Moderate	2
– Poor	1
– Degraded	0
**Osteochondral junction**	
– Normal	4
– Nearly normal	3
– Slight disruption	2
– Severe disintegration	1
– Disruption	0

*A score ranging from 0 to 4 points was assigned to each criterion.*

### Statistical Analyses

The results are expressed as means ± SEM of replicate determinations. Statistical analyses were performed using GraphPad Prism software (San Diego, United States). Comparative studies of means were performed by using the Mann-Whitney test when two conditions had to be compared and the Kruskal-Wallis test (*post hoc*: Dunn’s Multiple comparison test) when more than two conditions had to be compared.

## Results

### Rheological and Mechanical Properties

**FIGURE 1:**
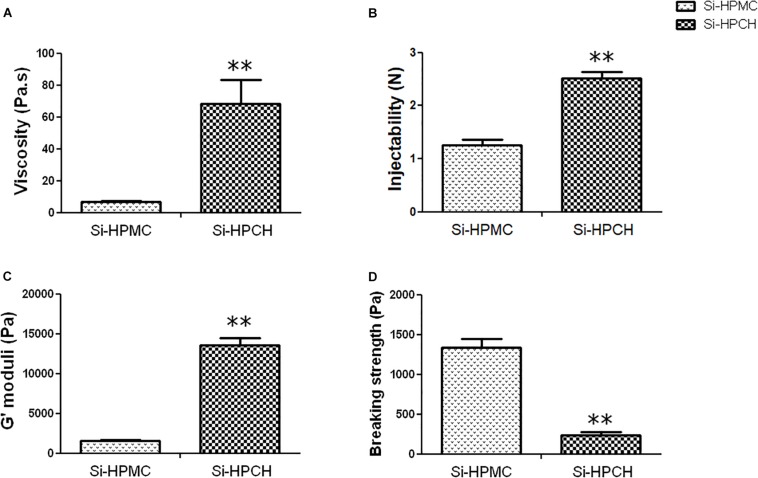
Rheological and mechanical.1pc properties of Si-HPMC and Si-HPCH hydrogels. As described in the section “Materials and Methods,” the viscosity **(A)** was measured with a MARS rheometer with a cone/plate geometry; the injectability **(B)** was investigated using a TAXT2 Texture Analyzer at an injectability rate of 17 sec per mL; the storage modulus **(C)** and the breaking strength **(D)** were measured with a MARS rheometer using a plate geometry. ^∗∗^*p* < 0.01 compared to the Si-HPMC condition (Mann-Whitney).

The objective was to develop a new hybrid hydrogel (Si-HPCH) by adding Si-Chitosan to an Si-HPMC hydrogel that was already being used for tissue repair. The formulation was characterized to determine its potential usefulness as a hydrogel for tissue engineering. Throughout the physicochemical characterizations, we evaluated the selected formulation for biological investigation. First, we measured the viscosity of the solutions. As reported in Figure 1A, the viscosity increased significantly when Si-chitosan was added compared to Si-HPMC alone. The viscosity profile of a solution is usually indicative of the injectability behavior. Nevertheless, the syringeability of the Si-HPCH solutions was monitored. As expected, the force required to inject increased when Si-chitosan was added, but the mixed solution remained readily injectable, with a value of 2.50 N (Figure 1B). Having evaluated the rheological properties of our hydrogels, we sought to investigate the mechanical properties of Si-HPCH. Its storage modulus (G′) and breaking strength (σ) were hence measured. The results, plotted in Figure 1C, show a 9-fold increase in G′ when Si-HPMC was combined with Si-Chitosan. In parallel, Figure 1D indicates that Si-HPCH exhibits a breaking strength 5.5-fold lower than that of Si-HPMC. Moreover, at the same concentration (4 wt%) of total polymer, Si-HPCH exhibited a 30% higher stiffness (G′) and a 30% decrease in the breaking strength.

### Cell Viability

To investigate the viability of ASC cultured in contact or embedded in Si-HPCH, we performed several *in vitro* and *in vivo* tests, (i) in 2D culture with an MTS-based metabolic assay, (ii) in 3D culture with a viability assay, and (iii) after subcutis implantation in nude mice.

**FIGURE 2:**
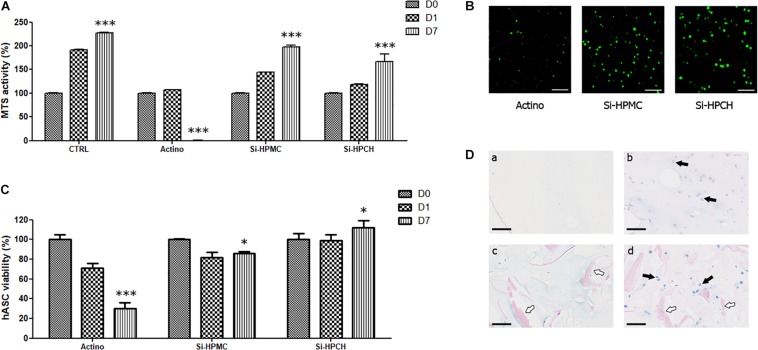
*In vitro* and *in vivo* viability of hASC. **(A)** MTS activity of hASC cultured in 2D. Cell viability was evaluated in 2D after molding Si-HPMC or Si-HPCH hydrogels on top of the cell layer (10,000 cells/cm^2^). As described in the section “Materials and Methods,” an MTS assay was performed on days 0, 1, and 7. The positive control (CTRL) was obtained by growing hASC alone, while the negative control was obtained by growing hASC in the presence of actinomycin D (Actino; 5 μg/mL). The results are expressed as the percentage of day 0 for each respective condition. ^∗∗∗^*p* < 0.001 compared to day 0 (Kruskal-Wallis). **(B,C)** 3D viability of hASC cultured in Si-HPMC or Si-HPCH hydrogels. Cell viability was evaluated in 3D after molding hydrogels mixed with 1 × 10^6^ hASC on days 0, 1, and 7 by the Live/Dead Cell Viability assay. The negative control was obtained by adding actinomycin-D (Actino; 5 μg/mL) to the culture medium. Pictures of representative samples of hASC into Si-HPMC cultured in the presence of actinomycin-D (Actino), into Si-HPMC (Si-HPMC) or into Si-HPMC (Si-HPCH) at day 7 are shown **(B)**. The scale bar represents 100 μm. Live and dead cells were then counted **(C)**. The results are expressed as the percentage of day 0 for each respective condition. ^∗^*p* < 0.05; ^∗∗∗^*p* < 0.001 compared to day 0 (Kruskal-Wallis). **(D)** *In vivo* hASC viability after subcutaneous implantation. 250 μL of Si-HPMC (panels a and b) or Si-HPCH (panels c and d) were implanted alone (panels a and c) or mixed with human ASC (panels b and d; 1 × 10^6^ cells/mL of hydrogel) in the subcutis of nude mice. After 6 weeks, explanted samples were histologically prepared for Alcian Blue staining. The back arrows indicate hASC while the white arrows indicate chitosan. The scale bar represents 100 μm.

The cytotoxicity of Si-HPCH hydrogel was first evaluated using hASC. As shown in Figure 2A, the MTS activity of hASC cultured in contact with Si-HPCH or Si-HPMC gradually increased from day 0 to day 7 (a 2-fold increase). After 7 days of culture, no difference in metabolic activity was observed between the three conditions (i.e., CTRL, Si-HPMC, and Si-HPCH). As expected, in the presence of actinomycin-D, the MTS activity decreased drastically from day 0 to day 7. Taken together, these data indicate that neither of the hydrogels resulted in a major alteration of the metabolic activity of 2D-cultured hASC. To further analyze whether Si-HPCH can influence the behavior of clinically relevant MSC, we also cultured hASC embedded three-dimensionally in Si-HPCH. Figure 2B illustrates the viability of hASC after 7 days of culture in 3D into Si-HPMC hydrogel in the presence of actinomycin-D (Panel Actino) or not (Panel Si-HPMC) and into Si-HPCH hydrogel (Panel Si-HPCH). Figure 2C shows the number of viable hASC when cultured three-dimensionally in Si-HPCH or Si-HPMC compared to cells cultured in Si-HPMC in the presence of actinomycin D.

Interestingly, a slight but significant increase was observed at day 7 for Si-HPCH, while a barely discernible decrease was observed for cells cultured in Si-HPMC. As expected, actinomycin-D induced a progressive decrease in hASC viability. Collectively, these data indicate that the addition of Si-chitosan to Si-HPMC did not significantly alter the viability of 3D-cultured hASC.

Finally, to determine whether Si-HPCH can support the viability of hASC *in vivo*, we mixed hASC (1 × 10^6^ cells/mL of hydrogel) with Si-HPMC or Si-HPCH prior to subcutis injection (250 μL) in nude mice. Both hydrogels were implanted alone as negative control. Six weeks after implantation, samples were retrieved and histologically analyzed. Macroscopically, no inflammation was observed in any condition. Interestingly, no cellular infiltration was observed in the Si-HPMC and Si-HPCH conditions as shown in Figure 2D (panels a and c).

To assess whether the embedded hASC could produce extracellular matrix components, we performed Alcian Blue staining to detect glycosaminoglycans (GAG). As indicated in Figure 2D (panels b and d), when the cells were implanted with Si-HPMC enriched with Si-chitosan (panel d), the cells produced a high amount of GAG as evidenced by the intense blue staining compared to cells implanted with Si-HPMC alone (panel b). Altogether, these *in vivo* results confirm the data generated by 2D and 3D culture and they highlight that Si-HPCH allows transplantation of viable hASC in the subcutis of nude mice while maintaining their viability and GAG-production activity for 6 weeks.

**FIGURE 3:**
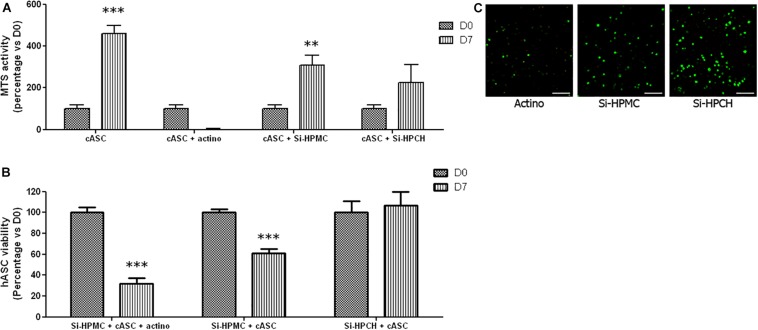
*In vitro* viability of cASC. **(A)** MTS activity of cASC cultured in 2D. Cell viability was evaluated in 2D after molding Si-HPMC or Si-HPCH hydrogels on top of the cell layer (10,000 cells/cm^2^). As described in the section “Materials and Methods” an MTS assay was performed on days 0 and 7. The positive control (CTRL) was obtained by growing cASC alone, while the negative control was obtained by growing cASC in the presence of actinomycin D (Actino; 5 μg/mL). The results are expressed as the percentage of day 0 for each respective condition. ^∗∗^*p* < 0.01; ^∗∗∗^*p* < 0.001 compared to day 0 (Kruskal-Wallis). **(B,C)** 3D viability of cASC cultured in Si-HPMC or Si-HPCH hydrogels. Cell viability was evaluated in 3D after molding hydrogels mixed with 1 × 10^6^ cASC on days 0, 1, and 7 by the Live/Dead Cell Viability assay. The negative control was obtained by adding actinomycin-D (Actino; 5 μg/mL) to the culture medium. Pictures of representative samples of cASC into Si-HPMC cultured in the presence of actinomycin-D (Actino), into Si-HPMC (Si-HPMC) or into Si-HPCH (Si-HPCH) at day 7 **(B)**. The scale bar represents 100 μm. Live and dead cells were then counted **(C)**. The results are expressed as the percentage of day 0 for each respective condition. ^∗∗∗^*p* < 0.001 compared to day 0 (Kruskal-Wallis).

Prior to embarking on a preclinical experiment of canine osteochondral defect repair with Si-HPCH and autologous ASC, we sought to check whether Si-HPCH can also support the viability of canine ASC. As shown in Figure 3A, we observed that the metabolic activity of cASC cultured in 2D while in contact with Si-HPMC or Si-HPCH increased from day 0 to day 7.

Whereas this increase was found to be statistically significant for cells cultured in contact with Si-HPMC, we failed to detect a significant increase in the metabolic activity with Si-HPCH. As expected, the MTS activity of cells cultured on a plastic substrate (CTRL) increased significantly, while that of cells cultured in the presence of actinomycin-D (Actino) dropped significantly from day 0 to day 7. To further document the effects of hydrogels on cell viability, we also analyzed the viability of cASC cultured 3D in Si-HPMC or Si-HPCH. Figure 3B illustrates the viability of cASC after 7 days of culture in 3D into Si-HPMC hydrogel in the presence of actinomycin-D (Panel Actino) or not (Panel Si-HPMC) and into Si-HPCH hydrogel (Panel Si-HPCH). As shown in Figure 3C, when cASC were 3D-cultured in Si-HPMC, there was a significant decrease in viability. Notably, cells cultured in Si-HPCH did not exhibit a decrease in cell viability. Viewed together, these data strongly suggest that Si-HPCH is a 3D-supportive scaffold for cASC viability.

### Repair of Osteochondral Defects in Dog

Having demonstrated that enrichment of Si-HPMC with Si-chitosan yields a mechanically reinforced hydrogel that is able to support the viability of canine and human ASC, we wanted to test the ability of this newly developed hydrogel to regenerate articular cartilage in a canine model of osteochondral defects. Osteochondral defects (6 mm in diameter and 5 mm in depth) were created in the knees of adult dogs and filled with Si-HPCH in the absence or presence of autologous ASC. All of the animals survived and fully recovered from the surgery. At 4 months after the surgery, the dogs were euthanized and condyles isolated for histological analyses of tissue repair. Macroscopic observation of the condyles revealed that one sample from the Si-HPCH/cASC group exhibited a fracture at the implanted site. This sample was, therefore, excluded from the study.

**FIGURE 4:**
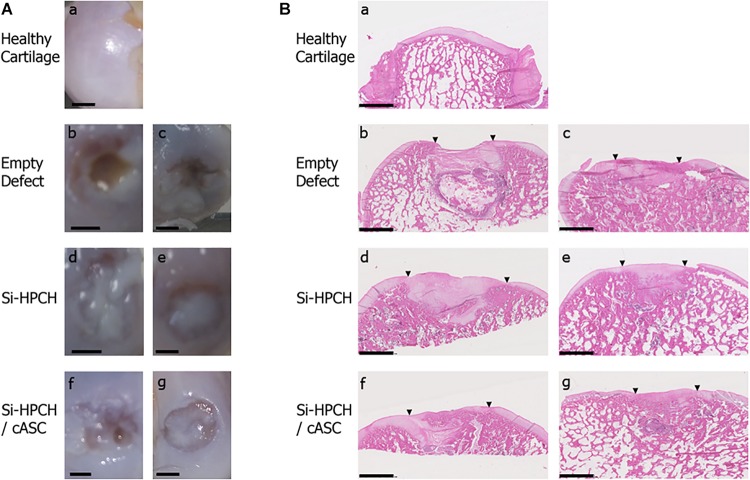
Macroscopic observation and histological characterization of tissue repair. Canine osteochondral defects (6 mm × 5 mm) were performed as described in the section “Materials and Methods.” The defects were either left empty (“empty” defects) of filled with Si-HPCH alone (“Si-HPCH”) or Si-HPCH associated with cASC (“Si-HPCH/cASC”). After 4 months of implantation, the dogs were sacrificed, the knees were retrieved, and the samples were macroscopically observed **(A)** or histologically characterized after hematoxylin-eosin-safran staining **(B)**. For each tested condition, the worst (b,d,f) and the best (c,e,g) repair outcomes are presented. Arrowheads indicate the edges of the defect. Healthy cartilage (a) of intact canine condyle is shown as an internal comparator. The scale bar represents 2.5 mm.

As illustrated in Figure 4, macroscopic observation (Figure 4A) of the samples did not reveal any abnormal findings indicative of inflammation. To further compare the histological features of the repair tissues, we then performed HE staining (Figure 4B). Figure 4A illustrates the worst (panels b, d, and f) and the best (panels c, e, and g) cartilage defect repair outcomes for each group compared to healthy cartilage (panel a). Notably, while there was barely any detectable repaired tissue in the empty defects, a newly formed tissue, partially covering the entire surface of the defects, was found in all the filled defects. Figure 4B illustrates the worst (panels b, d, and f) and the best (panels c, e, and g) cartilage defect repair outcomes for each group compared to healthy cartilage (panel a). These analyses of the repair tissue in the empty defect group (Figure 4B, panel b and c) revealed the presence of a fibrous tissue that was unable to induce an ad integrum covering of the defects.

**FIGURE 5:**
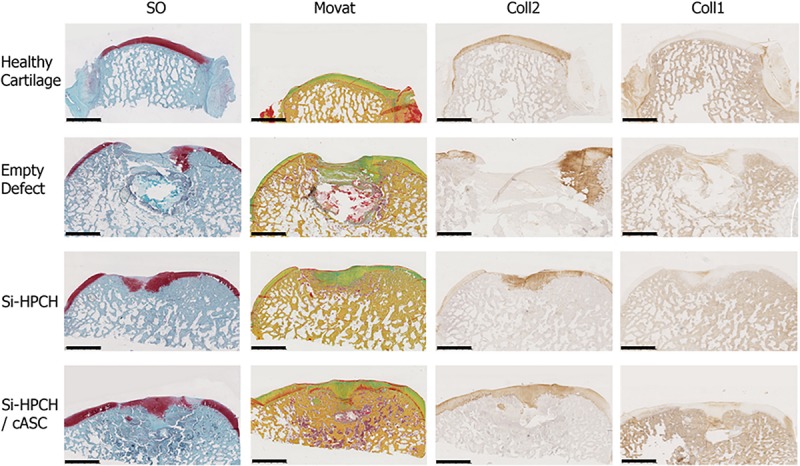
Extracellular matrix-specific histological and immunohistological analyses of tissue repair. Canine osteochondral defects (6 mm × 5 mm) were performed as described in the section “Materials and Methods.” The defects were left empty (“empty” defects) or filled with Si-HPCH alone (“Si-HPCH”) or Si-HPCH associated with cASC (“Si-PHCH/cASC”). After 4 months of implantation, the dogs were sacrificed, the knees were retrieved, and samples were histologically characterized by Safranin O (SO) and Movat pentachrome (Movat) staining. The samples were also immunostained for type II collagen (“Coll2”) and type I collagen (“Coll1”). Healthy cartilage of intact canine condyle is shown as an internal comparator. Representative histological slides are presented. The scale bar represents 2.5 mm.

To more specifically characterize the repaired tissue, histological (Safranin O and Movat pentachrome staining) and immunohistological analyses (type I and II collagen) were then performed (Figure 5). The repair tissue in the empty defect group (panel Empty Defect) was Safranin O-negative, thus indicating the absence of GAG, and did not exhibit any positivity for the immunodetection of type II collagen. A faint but detectable positive immunostaining for type I collagen was also observed, thereby confirming the fibrous nature of the repaired tissue. The defects treated with Si-HPCH or Si-HPCH/ASC were almost completely covered (Figure 5, panel Si-HPCH and Si-HPCH/ASC). Si-HPCH alone was found to induce an almost complete covering of the defects, while Si-HPCH associated with cASC, induced an ad integrum filling of the defects. In both conditions, the repaired tissue stained positively for the presence of GAG (Figure 5, SO) and collagen (Figure 5, Movat). Movat staining also revealed substantial bone remodeling in the subchondral area. Interestingly, in both conditions, the repaired tissue exhibited strong and diffuse immunostaining for type II collagen (Figure 5, Coll2) while remaining negative for type I collagen (Figure 5, Coll1). Altogether, these data very much suggest the production of a repaired tissue with cartilaginous features including the presence of GAG and type II collagen and the absence of type I collagen.

**FIGURE 6:**
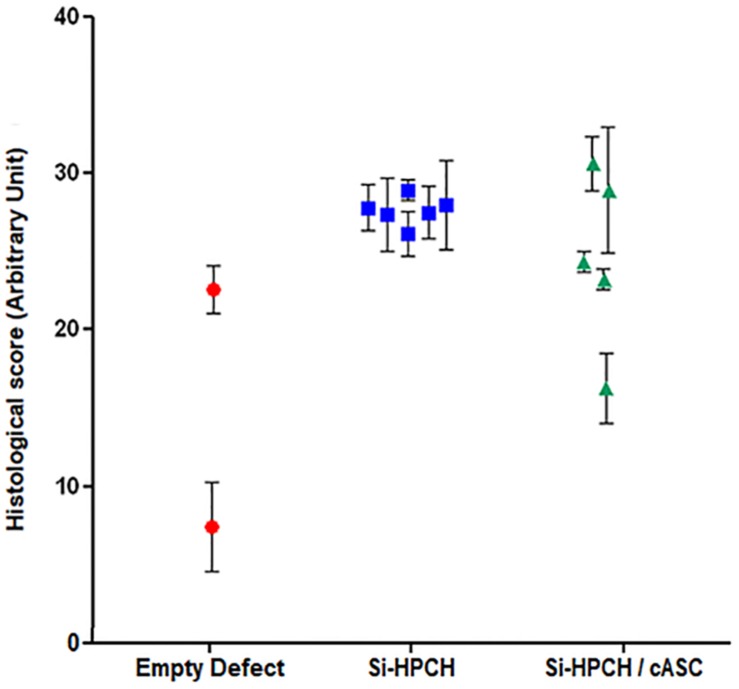
O’Driscoll-based quantitative scoring of cartilage repair. Canine osteochondral defects (6 mm × 5 mm) were performed and the defects left empty (“empty” defects) or filled with Si-HPCH alone (“Si-HPCH”) or Si-HPCH associated with cASC (“Si-HPCH/cASC”). After 4 months of implantation, the dogs were sacrificed, the knees were retrieved, and samples were histologically characterized (see Figure 5). An O’Driscoll-based histological scoring was then performed according to 10 histological parameters as described (Table 1). Each parameter was scored 0 to 4, with 4 being the best score. The results are expressed as individual plots for each condition.

Finally, to quantitatively assess the formation of a repaired tissue in the different conditions, HE staining (Figure 4) in association with the macroscopic appearance as well as SO, Movat, and anti-type I/II collagen staining (Figure 5) were used to provide a modified O’Driscoll scoring (Figure 6). Three independent assessors blindly scored the various samples. For the filled conditions, the O’Driscoll-based repair scoring (Figure 6) indicated that the Si-HPCH samples were more consistently repaired than the Si-HPCH/ASC-treated defects. Indeed, the mean score of the Si-HPCH samples was 27.6 ± 2.82 while for the Si-HPCH/cASC samples the mean score was 22.31 ± 8.14. Unfortunately, and probably because of the reduced number of samples, no statistical difference was recorded between the different groups, even though a clear tendency was noted.

## Discussion

In this study, we showed in a dog model of osteochondral defects that it is possible to repair articular cartilage with a hybrid hydrogel made of cellulose- and chitosan-derived polymers. After having shown that associating Si-HPMC with Si-chitosan led to the formation of a mechanically reinforced hydrogel that can support MSC viability *in vitro* and *in vivo*, we were able to clearly demonstrate the ability of this novel self-setting hydrogel to contribute to the repair of osteochondral defects in a canine model in the absence or presence of MSCs.

Due to the limited ability of cartilage to heal, untreated joint cartilage damage leads to progressive tissue degeneration that drastically increases the risk of the development of osteoarthritis (Clouet et al., 2009). It has been estimated that patients with cartilage lesions have a 5-fold increased risk of osteoarthritis (Gelber, 2000). The development of new therapies in cartilage regenerative medicine is, therefore, an issue of considerable importance. In order to devise treatments for cartilage damage, strategies combining cells and biomaterials have been developed. As a result of their physicochemical and biological properties, hydrogels are presently considered to be ideal candidates for cartilage tissue engineering. Over the past decade, our team has developed a hydrogel made of cellulose, referred to as Si-HPMC (Bourges et al., 2002a, b). Previous studies have shown that this Si-HPMC hydrogel is an injectable, self-setting, and biocompatible hydrogel that allows MSCs to regenerate soft tissues such as heart muscle (Mathieu et al., 2012) and colon (Moussa et al., 2017). As bone and joint tissues are chronically exposed to high mechanical stress, hydrogels need to have exceptional mechanically properties if they are to be suitable for repair of these tissues. In addition, in light of the role of ECM stiffness and rigidity on stem cell fate (Engler et al., 2008; Discher et al., 2009), it has become clear that synthetic ECM, such as hydrogels used to guide tissue regeneration, have to exhibit biomechanical properties that largely mimic those of the native tissue. In this context and considering the limited mechanical properties of Si-HPMC, we hypothesized that the addition of silanized chitosan to Si-HPMC could be a way to improve its mechanical properties. Interestingly, it has also been widely demonstrated that mechanical loading also influences the chondrogenic activities of MSC (Panadero et al., 2016; Choi et al., 2018). It could have been interesting to address whether combining mechanical loading and our Si-HPCH hydrogel may be a relevant strategy to improve MSC chondrogenesis.

In addition, chitosan was also considered due to its structural similarity to GAGs, which are a major component of the extracellular matrix of cartilage. Chitosan has been used for many years as a dressing and has been shown to improve wound healing with a high level of collagen deposition (Hamilton et al., 2006). Furthermore, like some GAGs such as hyaluronic acid, chitosan contains N-acetylglucosamine chains. As a result of this characteristic, chitosan may exhibit some of the biological activities of GAGs such as their ability to bind growth factors or their mucoadhesive properties (Martins et al., 2014). Therefore, this study aimed to improve the properties of an already developed Si-HPMC-based hydrogel by addition of Si-Chitosan as an additive within its network.

Moreover, previous studies have suggested that the mechanical properties of Si-HPMC remain far from those of native cartilage tissue. The stiffness of hydrogels is often reported has being two orders of magnitude lower than cartilage’s (100–1000 kPa) (Levental et al., 2007; Boyer et al., 2018). These low mechanical properties have drastically limited their clinical translatability and hydrogels are mainly used as space-filling scaffolds used for the delivery of bioactive molecules and cells (Drury and Mooney, 2003; Kopeček, 2007). To address this issue, we also focused our efforts on the development of a mechanically reinforced hydrogel.

In light of this, the first objective of this study was to characterize the mechanical properties of the hybrid Si-HPCH hydrogel that we had developed. We found that the addition of silanized chitosan to Si-HPMC increased the viscoelastic storage modulus G′ (nearly 10-fold). Interestingly, while the mechanical properties of Si-HPCH were determined to be higher than those of Si-HPMC, the Si-HPCH remained manually injectable, as indicated by its injectability force of less than 80 N (Vo et al., 2016). The increase in the viscosity and the storage modulus can be explained by the higher polymer concentration within the hybrid hydrogel (4 wt% for Si-HPCH compared to 2 wt% for Si-HPMC hydrogel). Shear mechanical results, storage modulus, and breaking strength, with the same Si-HPMC and Si-HPCH concentration of 4%, revealed an effect of the chitosan backbone on the mechanical properties of the hydrogel, with an increase of the G′ (8 vs. 14 KPa *p* < 0.05, data not shown) and a decrease in the breaking stress (1,300 vs. 800 Pa, *p* < 0.05, data not shown). However, the influence of the chitosan backbone on the rheological and mechanical properties of the hydrogel was not investigated throughout this study. These data clearly demonstrate that Si-HPCH, while exhibiting improved mechanical properties, remains manually injectable and can hence be used for mini-invasive surgical purposes.

In addition to their mechanical properties, the cytocompatibility of newly developed hydrogels is also a basic requirement for their use in tissue engineering. Since it is well known that improvement of the mechanical properties of a hydrogel often results in a decrease in its cytocompatibility, we performed an in-depth analysis of the cytocompatibility of Si-HPCH with respect to MSC. This observation can be explained by an increase in shear forces, as suggested by several studies (Gefen et al., 2008; Hong et al., 2016).

We first addressed whether MSC from human adipose tissue retain their viability when cultured in 2D or 3D while in contact with or embedded in Si-HPCH. The choice of MSCs was guided by the increasing use of these cells in regenerative medicine and tissue engineering studies (Ruiz et al., 2016). Indeed, although chondrocytes were the first cell type used for cartilage regenerative medicine, these cells exhibit many disadvantages, such as morbidity of the donor site and their *in vitro* dedifferentiation (Benya and Shaffer, 1982). These shortcomings led researchers to find a new source of cells for tissue engineering. In this context, MSCs are an attractive option because they can readily be harvested from different tissues [e.g., bone marrow (Friedenstein et al., 1968), adipose tissue (Fraser et al., 2006), etc.,]. In addition, MSCs have the capacity to differentiate into the chondrogenic lineage and to exhibiting immunomodulatory properties (Tyndall, 2015) that are clinically relevant for cartilage tissue engineering. Surprisingly, and while cell-cell contact have been largely proposed as a necessary prerequisite for MSC chondrogenesis (DeLise et al., 2000), our data strongly suggest that MSC embedded into a polysaccharidic hydrogel without any external pro-chondrogenic factors, may conserve their ability to commit into the chondrogenic lineage. Whether these data may be exploited to propose a cell-free approach for the repair of osteochondral defects now deserve to be further investigated.

The results obtained in our various viability tests indicate good cytocompatibility of Si-HPCH with respect to ASC (of human or canine origin) cultured either in 2D or 3D. To complement these *in vitro* viability assays, we also performed implantation of Si-HPCH associated with hASC in subcutaneous sites of nude mice. This *in vivo* experiment confirmed that Si-HPCH was able to support the viability of hASC even after 6 weeks of *in vivo* implantation. Interestingly, our histological analyses of samples retrieved from the injection site also indicate that hASC, when implanted with Si-HPCH, exhibited a notable ability to produce GAG. Whether this ability of cells to produce ECM component when embedded in chitosan-containing cellulose hydrogel is related to the biological properties of chitosan, including its ability to interact with growth factors (Raftery et al., 2013), warrants further exploration.

Finally, to assess whether Si-HPCH may be a good candidate for cartilage repair, we undertook animal testing in dog, as they remain a clinically relevant veterinary target for cartilage repair strategies. Dogs are commonly affected by joint dysplasia (Ytrehus et al., 2007; Pascual-Garrido et al., 2018; Soo et al., 2018) and there is still not an effective therapeutic treatment for the resulting osteochondral defects. As is the case in humans, these untreated osteochondral defects often lead to an increased risk of osteoarthritis and severe disability.

The results of our preclinical testing in dogs showed that, 4 months after the implantation of hydrogels in osteochondral defects, Si-HPCH allowed for an almost complete filling of the osteochondral defects irrespective of the presence of autologous ASC.

Histological and immunohistological analyses of the newly formed tissue indicated the presence of a hyaline cartilage-like tissue with a GAG- and type II collagen-rich ECM. While these analyses are evidence for the presence of a tissue resembling healthy articular cartilage, several *in situ* biomechanical analyses can now be performed to further document the cartilaginous nature of this repaired tissue.

Unexpectedly, our data also revealed that defects filled with Si-HPCH alone exhibited a degree of repair that was quite comparable to what was observed for defects filled with Si-HPCH/ASC. Of particular interest for the clinical translation of our data, O’Driscoll scoring revealed that defects treated with Si-HPCH alone had a highly consistent and reproducible repair process while those treated with Si-HPCH/ASC exhibited a greater degree of inter individual variability. These data, which are presumably related to the great variability of ASC properties between individuals, are likely to raise some important issues regarding the ability to exploit the regenerative properties of ASC. The ability of Si-HPCH to support cartilage repair also raises the possibility that this hydrogel, due to the growth factor interacting properties of its chitosan moieties, may promote chemoattraction of regenerative cells emanating from subchondral bone marrow. This hypothesis, although appealing due to the clinical potential, should, however, be tested further before definitive conclusions are drawn.

To conclude, we have developed a new hybrid hydrogel made of silylated chitosan and HPMC that has better mechanical properties, hand-compatible injectability, and *in vitro* and *in vivo* cytocompatibility. This Si-HPCH hydrogel supports the repair of load-bearing osteochondral defects in a canine model in the presence or absence of adipose stromal cells.

The possibility of using Si-HPCH for the development of a cell-free repair strategy of osteochondral defects may be of great value for cartilage clinicians, notably by reducing cell dependent inter-individual variability, cost, logistical complexity, and regulatory concerns. Testing of its efficacy in canine or equine patients with naturally occurring and OA-related osteochondral defects should now be undertaken to further address the clinical value of Si-HPCH in cartilage repair.

## Data Availability Statement

The raw data supporting the conclusions of this article will be available by the authors, without undue reservation, to any qualified researcher.

## Author Contributions

CB, PW, CV, OGe, OGa, and JG contributed to conception and design of the study. CB, GR, Cd’A, JL, CV, BH, OGe, MF, GV, PR, and OGa carried out the experiments. CB performed the statistical analysis. CB, GR, PW, OGa, and JG wrote sections of the manuscript. All authors contributed to the manuscript revision, read and approved the submitted version.

## Conflict of Interest

The authors declare that the research was conducted in the absence of any commercial or financial relationships that could be construed as a potential conflict of interest.
